# Outcome of Pregnancies After Balloon Occlusion of the Infrarenal Abdominal Aorta During Caesarean in 230 Patients With Placenta Praevia Accreta

**DOI:** 10.1007/s00270-016-1418-y

**Published:** 2016-07-20

**Authors:** Qinghua Wu, Zhuan Liu, Xianlan Zhao, Cai Liu, Yanli Wang, Qinjun Chu, Xiaojuan Wang, Zhimin Chen

**Affiliations:** 1 Departments of Prenatal Diagnosis, and Obstetrics, Obstetric Critical Treatment Center of Henan Province, The First Affiliated Hospital of Zhengzhou University, Jian She Dong Lu, No 1, Zhengzhou City, Henan Province China; 2 Obstetric Critical Treatment Center of Henan Province, Department of Obstetrics, The First Affiliated Hospital of Zhengzhou University, Jian She Dong Lu, No 1, Zhengzhou City, Henan Province China; 3 Department of Anesthesiology, The First Affiliated Hospital of Zhengzhou University, Jian She Dong Lu, No 1, Zhengzhou City, Henan Province China

**Keywords:** Placenta praevia accreta, Balloon occlusion of the infrarenal abdominal aorta, Caesarean

## Abstract

**Purpose:**

To explore the efficacy and safety of prophylactic temporary balloon occlusion of the infrarenal abdominal aorta during caesarean for the management of patients with placenta praevia accreta.

**Methods:**

Two hundred and sixty-eight cases of placenta praevia accreta from January 2012 to June 2015 were retrospectively reviewed. Group A included two hundred and thirty patients who underwent prophylactic temporary balloon occlusion of infrarenal abdominal aorta followed by caesarean section. Group B included thirty-eight patients who underwent caesarean without endovascular intervention. The parameters including operating room time, estimated blood loss, blood transfusion volume, PT (prothrombin time) during operation, days in the intensive care unit, and total hospital days were compared between the two groups.

**Results:**

The operating room time, estimated blood loss, PT, the incidence of hysterectomy, blood transfusion volume, postpartum haemorrhage, and days in intensive care unit were lower in group A than in group B, with statistical significance (*P* < 0.05). There was no significant difference in the Apgar scores of the neonates and the incidences of thrombosis in lower limbs between the two groups (*P* > 0.05). No patient in the group with prophylactic temporary balloon occlusion of the infrarenal abdominal aorta was performed hysterectomy, while three patients in group B were performed hysterectomy because of uncontrollable haemorrhage.

**Conclusions:**

The results indicate that prophylactic temporary balloon occlusion of infrarenal abdominal aorta followed by caesarean section is safe and effective to control intraoperative blood loss and greatly decreases the risk of hysterectomy in patients with placenta praevia accreta.

## Introduction

Placental disorders such as placenta previa, placenta accreta, and vasa previa are all associated with vaginal bleeding in the second half of pregnancy, which are also important causes of serious foetal and maternal morbidity and also mortality. The rates of previa and accreta are increasing [1] with the rising incidence of caesarean section, especially in China, with the rate of caesarean being close to 50 % and, in some cities, even more than 70 %. Placenta praevia is an obstetric complication in which the placenta is attached over the lower uterine segment. Placenta accreta is a disorder of placental implantation characterized by penetration of the placental villi into the uterine wall or even into the adjacent organs, which is subdivided into three distinct entities based on the depth of placental invasion. Placenta accreta is the least invasive form in which the ingrowth of the placental villi occurs through the thinned decidua basalis and the layers of Nitabuch. The term placenta increta is characterized by the invasion of the placenta into the myometrium. Invasion through the myometrium reaching or penetrating the serosa is termed placenta percreta. It is widely accepted that the most important risk factor for placenta accreta is the increasing rate of caesarean section, particularly the co-existing placenta praevia [2], which may be caused by abnormal decidualization of a scarred area or lack of decidua in the lower uterine segment near the cervix [3]. Placenta praevia accreta is a major cause of caesarean hysterectomy with potential risk of maternal mortality and morbidity, which is among the greatest treatment challenges in modern obstetrics.

Antepartum diagnosis of placenta accreta can be made by pelvic ultrasound or MRI, which is very useful for the obstetrician to make preparations for delivery of the neonate, and safe removal of the placenta with a minimal risk of postpartum haemorrhage. Hysterectomy following caesarean is a conventional treatment for placenta praevia accreta. Other surgical options have also been performed to minimize the blood loss, including proximal ligation of the internal iliac artery or uterine artery and embolization of internal iliac artery or uterine artery. The efficacy of occlusion of internal iliac artery or uterine artery is limited by rapid recruitment of an extensive collateral system in the pelvis. The abdominal aorta is an alternative site proposed for occlusion.

The effective value of prophylactic balloon occlusion of the infrarenal abdominal aorta is not that clear because of limited amount of literature and cases. Since 2012, in our group, prophylactic temporary balloon occlusion of the infrarenal abdominal aorta during caesarean has begun to be performed in the management of patients with placenta praevia accreta. In this study, the clinical data of 268 cases of placenta praevia accreta were retrospectively analysed; 230 of them underwent prophylactic infrarenal abdominal aortic balloon occlusion; and all the patients were avoided hysterectomy. We aimed to discuss the optimal operation modalities for this disease.

## Materials and Methods

### Patients

Our Institutional Ethical Committee approved this study protocol. Written informed consent was obtained from all the patients included in this study. From January 2012 to June 2015, a total of 268 pregnant women with placenta praevia accreta diagnosed by ultrasound or MRI were recruited in this study. Group A included 230 patients who accepted the prophylactic infrarenal abdominal aortic balloon occlusion. Group B included 38 patients who did not go for prophylactic infrarenal abdominal aortic balloon occlusion because 18 of them were hospitalized between January and April in 2012 during which period the digital subtraction angiogram (DSA) operation room was not available. Balloon occlusion could not be performed in the majority among other 20 patients since they were referred to this hospital in emergency or the DSA operation room was occupied, alternatively selected to get the traditional operation.

There was no significant difference at age, gravidity, parity, and gestational age at delivery between both groups (see Table 1).

**Table 1:** Demographic and obstetric characteristics of patients

Group	Total	Age (years)	Gestation (weeks)	Gravidity (*n*)	Parity (*n*)
A	230	29.5 ± 3.6	35.6 ± 1.3	4.2 ± 1.1	2.0 ± 0.6
B	38	30.4 ± 4.0	35.5 ± 1.5	3.9 ± 1.0	2.1 ± 0.7
*t* test	–	1.212	0.533	1.414	0.821
*P* value	–	0.228	0.595	0.160	0.413

### Methods

**Fig. 1:**
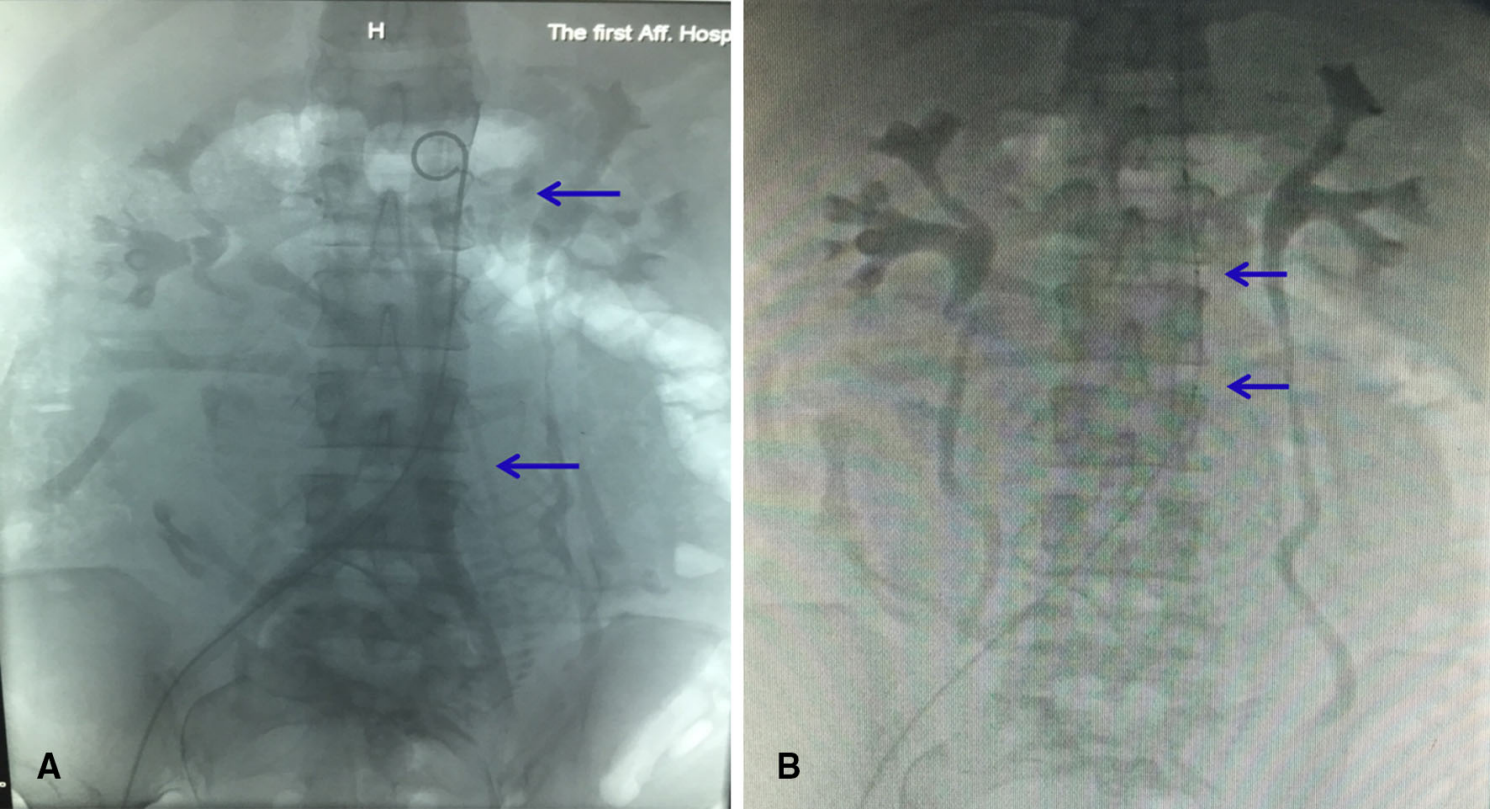
**A** The upper *blue* *arrow* points to the renal artery which was located in the middle of the lumbar 1 vertebral body shown by abdominal aortic angiography; the lower *blue arrow* points to iliac artery bifurcation at the upper edge of the 4 lumbar vertebral body. **B** The catheter was prophylactically placed in the infrarenal artery, and the two *blue arrows* point to both ends of the balloon

**Fig. 2:**
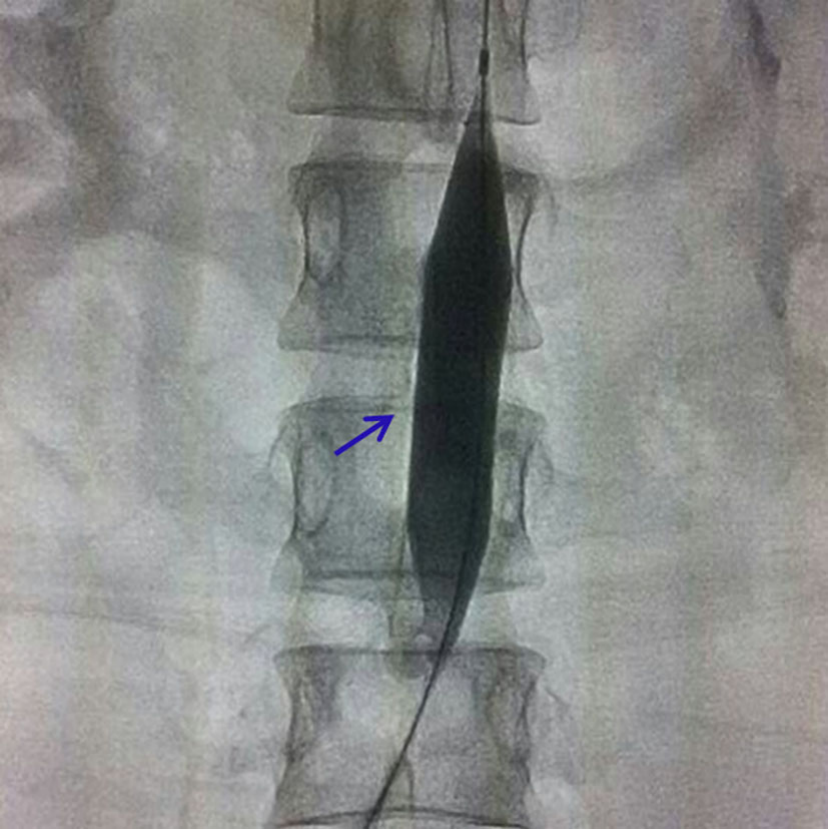
Balloon was inflated to occlude the infrarenal artery, see *blue* *arrow*

The balloon occlusion of the abdominal aorta was performed on all the hemodynamically stable patients by the interventional radiologist in DSA operating room under local anaesthesia immediately before caesarean section. The diameter of the abdominal aorta was measured by ultrasound or MRI, and the proper type of balloon was selected by interventional radiologists. The origin of renal artery and iliac artery bifurcation can be clearly shown by abdominal aortic angiography (Fig. 1A). Using a standard Seldinger technique, the 5 F pigtail occlusion balloon catheter (Cook, Bloomington, IN, USA) introduced through an 8-F sheath (Cook) was inserted from the right femoral artery to the infrarenal abdominal aorta at the level of T12, and 5 ml of iodixanol (Visipaque-320, Nycomed, Oslo, Norway) was injected to locate the origin of the renal arteries (Fig. 1B) and be fixed carefully. An 8-F, 40 mm × 16 mm or 40 mm × 18 mm balloon catheter (Bard Peripheral Vascular, Tempe, AZ, USA) was inserted into the abdominal aorta. Inflated balloon was placed under the origin of the renal arteries. Upon foetal delivery and umbilical cord clamping, the occlusion balloon was inflated to the size needed to occlude the aorta using 10–16 ml of saline solution (Fig. 2). Complete occlusion was deemed to have been achieved when the patient’s pulse and oxygen saturation could no longer be monitored using the pulse oximeter, and when the curve of the blood pressure sensor presented an approximately straight line. The reduction of blood flow obtained by inflating the balloon showed the benefit of making the surgical field less bloody. The balloons were inflated for 12–15 min, and the inflations were alternated with deflations of 1–2 min. After the operation, pelvic angiography was performed again. If there was active bleeding, uterine artery embolization (UAE) was supplemented. The fluoroscopy time was recorded in all the cases. When the operation was completed, the catheter was pulled out and compression bandaging of femoral artery puncture sites was performed. The lower limbs of the patients were given massage after operation. Low-molecular-weight heparin was applied to the patients in group A after 24 h to prevent vein thrombosis of lower limbs.

In group B, patients were given caesarean section without balloon occlusion of abdominal aorta. In both groups, according to the placenta accreta location and depth, patients were given local excision of uterine wall, placenta evacuation, partial cystectomy, and bladder repair. 20 U of oxytocin and 250 µg of Tromethamine were injected in the myometrium. Conservative treatments in placenta accreta including oversewing the placental bed, uterine tamponade, and bilateral uterine artery ligation were used. Hysterectomy was performed when the bleeding could not be controlled.

Estimated blood loss was measured with reference to the contents of the suction jar in the operating room and to the weight of the surgical pads.

The balloon inflation time was recorded. The foetal radiation dose during the operation was approximately calculated as the entrance skin radiation dose in the area of the irradiated field. The exact fluoroscopy time and radiation dose were supplied by the fluoroscopy machine at the end of the procedure.

SPSS 17.0 software was used to do the statistics. The results were presented as mean ± standard deviation values and compared by using the independent samples *t* test. The comparison of nonparametric values was accomplished by using Fisher’s exact test or Chi square test. Statistical significance was set at *p* < 0.05.

## Results

In both groups, diagnosis of placenta praevia accreta was validated by the intraoperative finding. Placenta accreta or increta was confirmed in patients when manual removal of the placenta was impossible because of the absence of complete cleavage plane between the placenta and the uterus. Placenta percreta was confirmed in patients based on the presence of a disjunction of the myometrium in association with bladder involvement at caesarean section. The majority of the patients in this study were grouped into placenta increta and percreta: 49 % (112/230) of the patients in group A as increta and 38 % (88/230) as percreta, and 45 % (17/38) of the patients in group B as increta and 39 % (15/38) as percreta. There was no significant difference in the degree of placenta implantation in both groups (Table 2).

**Table 2:** Statistical comparison of the position of praevia placenta observed during operation

Group	Total	Partial	Myometrium penetrated	Posterior bladder wall involved	Posterior bladder wall penetrated
A	230	30	112	59	29
B	38	6	17	10	5

*X* ^2^ = 0.303, *P* = *0.960*

Hysterectomy was required in three of the 38 patients in group B because of intractable bleeding. All cases in group A were successfully treated without performing hysterectomy. Median estimated blood loss during caesarean section was 921 ± 199 and 2790 ± 335 ml in group A and group B, respectively, with a significant statistical difference. Median blood transfusion volume in Group A (422 ± 58 ml) was less than that in Group B (1580 ± 67 ml) with a significant statistical difference. No difference in blood loss was found in either group after the operation. Patients in group A presented a significant advantage compared to group B in terms of operating time (64.1 ± 5.1 vs 92.1 ± 9.7 min, *p* < 0.0001), admission to ICU (*p* < 0.0001), and postoperative stay (*p* < 0.0001). The average prothrombin time (PT) in group B was longer than that in group A (*p* < 0.0001), see Tables 3 and 4. Two patients with placenta percreta in group A were performed temporary aortic balloon occlusion followed by UAE. One case with severe haemorrhage in Group B was given UAE and avoided of hysterectomy. Bilateral UAE was performed with gelatin sponge particles of 710–1000 µm (Alicon Pharm SCT&TEC, Hangzhou, China), which were soaked in iodixanol and injected until no active bleeding was observed.

**Table 3:** Intraoperative data in the two groups

Group	Cases	Operating duration (min)	Intraoperative blood loss (ml)	Blood transfusion volume (ml)	Hemodynamical abnormality (decreased blood pressure or increased heart rate)	Hysterectomy
A	230	64.1 ± 5.1	921 ± 199	422 ± 58	9.2 ± 1.0	0 (0/230)
B	38	92.1 ± 9.7	2790 ± 335	1580 ± 67	12.2 ± 1.1	7.89 (3/38)
*t* test	–	16.345	30.67	15.18	12.801	–
*P* value	–	0.000	0.000	0.000	0.000	0.003

**Table 4:** Postoperative data in the two groups

Group	Cases	Admission to ICU	Stay in hospital after operation (day)	Vein thrombosis of lower limbs
A	230	1.30 (3/230)	5.1 ± 0.8	0.87 (2/230)
B	38	13.15 (5/38)	6.7 ± 1.0	0
*t* test	–	11.994	4.571	–
*P* value	–	0.001	0.000	1.000

In Group A, the average balloon inflation time in all the patients of group A was 23.6 ± 7.8 min, the mean radiation exposure time was 8.3 ± 3.9 s, and the average foetal radiation dose was 5.1 ± 3.0 mGy.

Apgar scores of the neonates at 1 min and 5 min were >8 in all delivered foetuses, without significant differences between the groups (*P* > 0.05). There was no significant difference in the birth weights among the newborns between the groups (*P* = 0.106), see Table 5.

**Table 5:** Comparison of the two groups on Apgar scores of the neonates and weight at birth

Group	Cases	1 min	5 min	Weight birth (kg)
A	230	8.8 ± 0.9	9.7 ± 0.5	2.7 ± 0.2
B	38	8.4 ± 0.9	9.6 ± 0.6	2.6 ± 0.2
*t* test	–	1.747	1.205	1.635
*P* value	–	0.084	0.232	0.106

There was no maternal or foetal mortality in either group, and all the mothers and babies were healthy at the time of discharge. Two patients in group A had vein thrombosis of lower limbs and recovered after conservative treatment. Of all the 197 patients in Group A available for follow-up, 105 patients have resumed normal menstruation, and sonography results showed no uterine abnormalities in 195 patients, but scar diverticulum in two patients (less than 1 cm). In group B, 30 patients were available to be followed up, of which 19 of them had normal menstruation then. No uterine abnormalities have been detected by transvaginal sonography.

## Discussion

The incidence of caesarean section operation has been increasing in China in the recent years, accompanying the increased complication of placenta praevia accreta. Early diagnosis together with advance planning of placenta praevia accreta is the key to minimize morbidity. The diagnosis is usually made by ultrasonography or MRI technique. Pelvic ultrasound is regarded as the most commonly used imaging modality for the diagnosis of placenta praevia accreta, and the ultrasound findings include the loss of the normal hypoechoic retroplacental myometrium zone, thinning or disruption of the hyperechoic uterine serosa–bladder interface, the presence of focal exophytic mass lesions, and the presence of lacunae in the placenta [4]. Three D power Doppler would have the best positive predictive value (76 %), followed by grey-scale (51 %) and colour Doppler (47 %) [5]. For the detection of placenta accreta, compared to the sensitivity of 0.77 and specificity of 0.96 by ultrasound, MRI has been shown to have a sensitivity of 0.88 and specificity of 1.0 [6]. Therefore, MRI has been applied as an adjunct in diagnosis when the ultrasound results are equivocal or inconclusive. In this study, most of the patients recruited in this study were performed both pelvic ultrasound and MRI examination, except for the patients present in emergency, who could not have enough time to be examined by MRI. All the patients included in this study were confirmed of the diagnosis of placenta praevia accreta by the intraoperative finding.

In the patients with placenta praevia accreta, the amount of bleeding is about 3000–5000 ml [7], even more than 10,000 ml in some situations. Several surgical options have been attempted to minimize blood loss in patients with placenta praevia accreta. The recommended treatment is hysterectomy [8], which is not easy to accept for most of the patients since it deprives the chance of fertility. Pelvic embolization including common iliac artery, internal iliac artery, or uterine artery has been used in the treatment of obstetric haemorrhage or postpartum haemorrhage with procedural success in 95 % of patients, who have been avoided of hysterectomy or severe blood loss [9, 10]. However, embolization may induce immediate or late adverse events, and uterine necrosis and ovarian failure are the classic complications. The latter was more preferred since the balloon may be inflated intermittently during the operation or may be left inflated if excessive bleeding occurs [11, 12]. Most of the studies have used the internal iliac artery, the anterior division of the iliac artery, or the uterine artery as sites for balloon occlusion in patients with praevia accreta [10, 11, 13–15], but all the reported cases were just in a small number and with high incidence of hysterectomy.

The internal iliac artery is the main blood supply to pelvic cavity and is divided into anterior and posterior divisions. The uterine artery, a branch of the anterior division, is the main supply to uterus. However, there are several other vascular territories that provide a rich collateral supply to the uterus. Therefore, the abdominal aorta is an alternative site proposed for occlusion, which may greatly diminish the collateral supply. Up to now, a total of less than ten literatures with very few patients using the infrarenal abdominal aortic position for balloon occlusion were reported, and the majority was eventually given caesarean hysterectomy [13, 16–20]. Unlike the internal iliac artery and common iliac artery where bilateral balloon placement is needed, the procedure of prophylactic infrarenal abdominal aortic balloon occlusion is relatively easy, as the balloon can be placed rapidly because a single catheter insertion is sufficient. There is no need for contrast injection and radiation exposure is minimal. Lower abdominal aortic balloon occlusion significantly reduces blood loss and offers more time for surgeons to perform. Therefore, prophylactic balloon occlusion of infrarenal abdominal aorta has more advantage than balloon occlusion of internal iliac artery or uterine artery.

In this study, prophylactic temporary balloon occlusion of infrarenal abdominal aorta was performed in 230 patients with praevia accreta providing better hemostatic results, the procedure time decreases, and no hysterectomy was needed. Only two cases with placenta percreta were also given intraoperative uterine artery embolization. The main complications with balloon occlusion of infrarenal abdominal aorta are distal ischemia, reperfusion injury, thrombosis, and embolization of the lower extremities. However, these complications can be avoided by limiting the occlusion time as much as possible. In this study, the balloon inflation time in all the patients of group A was less than 30 min (average 23.6 ± 7.8), and no complications were detected after the operation. In control group, patients were applied traditional operation, ligation of uterine arteries, and tourniquet application. The amount of bleeding and duration of operation were more than patients with prophylactic balloon occlusion of infrarenal abdominal aorta. In the study reported by Panici in 2012 [21], intraabdominal aortic balloon occlusion was adopted and hysterectomy was needed in 15 % (2/15) of the patients with placenta praevia accreta with significantly reduced blood loss. Compared with more previous literatures which comprise a small number of patients, this study included the largest number of cases of praevia accreta treated by prophylactic balloon occlusion of infrarenal abdominal aorta and showed satisfactory outcome.

Foetal radiation exposure is of great concern to all the patients, and limiting the fluoroscopy time is the most important step to reduce the radiation dose. Compared to prophylactic internal iliac artery balloon occlusion procedure, during which the average foetal radiation doses ranged from 21–61 mGy [11, 22–24], in this study, the mean exposure time was 8.3 ± 3.9 s and the average foetal radiation dose was 5.1 ± 3.0 mGy. Performing the angiographic and surgical procedures in the same DSA operation room may reduce the foetal exposure to radiation. Also, the procedure of prophylactic infrarenal abdominal aortic balloon occlusion is relatively easy, which may save operation time. According to the National Council on Radiation Protection and Measurements, the risk to a foetus from diagnostic imaging is significantly increased when the radiation exposure exceeds 150 mGy. Yet, at doses of less than 50 mGy, the risk for radiation-induced abnormality is considered negligible [25]. Therefore, exposure to X-rays for a short duration during the operation with prophylactic balloon occlusion of infrarenal abdominal aorta would be mostly safe for the foetuses.

Another team in our group [26] has shown that temporary aortic balloon occlusion followed by UAE for the patients with placenta praevia accreta can effectively control postpartum haemorrhage during placental dissection, which can also reduce transfusion requirements, hysterectomy rate, and operation time in patients. Among 42 patients, hysterectomy was only required in one patient. In this study, UAE was not commonly applied, only two patients in Group A and one in Group B with uncontrollable bleeding were given UAE and these two cases in group A were performed in 2013, while we just started temporary balloon occlusion of infrarenal abdominal aorta and were not that experienced. Since the good outcome of the patients performed prophylactic temporary balloon occlusion of infrarenal abdominal aorta followed by caesarean section and lower cost without UAE, now all the doctors in our group prefer prophylactic balloon occlusion of infrarenal abdominal aorta without routine application of UAE, except for uncontrolled bleeding.

In summary, based on our experience and comparison of different modalities in the treatment of placenta praevia accreta, we show that prophylactic temporary balloon occlusion of infrarenal abdominal aorta followed by caesarean section is an easy, safe, and effective option to control intraoperative blood loss and postpartum haemorrhage, and to decrease the risk of hysterectomy, thus preserving the fertility in patients with praevia accreta.
